# ﻿A new species of *Pleurothallis* in the *P. cardiostola*-*P. lilijae* complex of section Macrophyllae-Fasciculatae (Orchidaceae, Pleurothallidinae) from Ecuador and Peru

**DOI:** 10.3897/phytokeys.262.157111

**Published:** 2025-09-12

**Authors:** Fiorela Revatta-Bustos, José D. Edquén, Jessy P. Arista, Elmer Yrigoín, Rosalynn Yohanna Rivera López, Mark Wilson, Mabel Enco, Kely Edquen, Santos Leiva-Espinoza, Manuel Oliva-Cruz, Gerardo A. Salazar

**Affiliations:** 1 Facultad de Ingeniería Ambiental, de Biosistemas y de la Energía, Universidad Nacional Toribio Rodríguez de Mendoza de Amazonas, Chachapoyas, Amazonas, Peru; 2 Instituto de Investigación para el Desarrollo Sustentable de Ceja de Selva (INDES–CES), Escuela de Posgrado, Universidad Nacional Toribio Rodríguez de Mendoza de Amazonas, Chachapoyas, Amazonas, Peru; 3 Universidad Nacional de San Martín (UNSM), Tarapoto, San Martín, Peru; 4 Department of Organismal Biology and Ecology, Colorado College, 14 East Cache La Poudre, Colorado Springs, CO 80903, USA; 5 Universidad Católica Sedes Sapientiae - Filial Rioja, Nueva Cajamarca, San Martín, Peru; 6 Instituto de Investigación para el Desarrollo Sustentable de Ceja de Selva (INDES–CES), Universidad Nacional Toribio Rodríguez de Mendoza de Amazonas, Chachapoyas, Amazonas, Peru; 7 Departamento de Botánica, Instituto de Biología, Universidad Nacional Autónoma de México, Apartado Postal 70-367, 04510 México City, Mexico

**Keywords:** Área de Conservación Privada Huaylla Belén-Colcamar, Cajanuma Range, Podocarpus National Park, rural community of Diosan, montane cloud forests

## Abstract

A new species of Pleurothallis subgenus Pleurothallis section Macrophyllae-Fasciculatae, recently discovered in upper montane cloud forests of the province of Loja, southern Ecuador, and the department of Amazonas, northern Peru, is described and illustrated as *P. labajosii***sp. nov**. It is similar to other species of the *P. cardiostola–P. lilijae* complex, especially *P. alopex*, but differs from the latter in its oblong-lanceolate leaves; pale yellow to reddish-maroon, resupinate flowers; elliptic to elliptic-ovate, 5-veined dorsal sepal; ovate to ovate-triangular, 4-veined synsepal; recurved petals; and deep, triangular, minutely papillose labellum cavity with a deep, rounded glenion. A distribution map and information on the ecological preferences of the new species are provided.

## ﻿Introduction

Montane cloud forests are crucial biotic elements of the tropical Andes, as they are among the world’s ecosystems that hold the highest levels of taxonomic diversity and endemism (Myers et al. 2000; Kappelle 2004). They are significant because of their marked environmental heterogeneity, which creates complex ecological gradients that promote adaptation and specialization of species (Bruijnzeel et al. 2011). Within this ecological complexity, the Orchidaceae stand out as one of the most diverse and specialized plant groups, with a high percentage of endemic species adapted to particular ecological niches (Dressler 1993; Arista et al. 2023; Ackerman et al. 2025) . However, many species remain unknown to science because of insufficient botanical exploration, while there is an increasing threat caused by human activities such as deforestation, expansion of agriculture, and climate change (Gerstner and Zarnetske 2025; IPCC 2023).

The genus *Pleurothallis* R.Br. is one of the largest among the Neotropical orchids, currently with 551 to 713 described species, depending on synonymy (M. Wilson, unpublished, continuously updated database). These species occur in a variety of biomes from Mexico and Central America through the Caribbean region south to Bolivia and Paraguay in South America, with a high concentration of taxa in the Andes (Jiménez et al. 2021). This region sustains a high number of endemic species, a reflection of the ecological complexity of the region and its vast floristic diversity (Jiménez et al. 2018; Ocupa-Horna et al. 2023; Baquero et al. 2024).

Within *Pleurothallis*, subgenus Pleurothallis section Macrophyllae-Fasciculatae Lindl. (1859) is characterized by its slender stems (“ramicauls”) with a usually ovate to lanceolate leaf typically with a cordate base and an abbreviate, compound inflorescence producing several single-flowered coflorescences, often with a single flower open at a time (see Rojas-Alvarado and Karremans 2024 for inflorescence terminology). The lateral sepals are fused into a synsepal similar to the dorsal sepal, on which the lip is placed, and the column is short, with a bilobed stigma and two pollinia (Lindley 1859; Luer 2005; Wilson et al. 2016; Sierra-Ariza et al. 2022). A preliminary phylogenetic analysis by Wilson et al. (2011) showed that section Macrophyllae-Fasciculatae forms a well-defined clade within *Pleurothallis* and is the most species-rich group within the genus, with an estimated 276 to 347 described species, depending on the author, and its known diversity continues to expand with the discovery and description of new species (Wilson et al. 2016, 2022; Sierra-Ariza et al. 2022).

In the present work, an additional species in the *Pleurothallis cardiostola* Rchb.f.–*P. lilijae* Foldats complex of section Macrophyllae-Fasciculatae, discovered recently in montane cloud forests in southern Ecuador and northern Peru, is described, illustrated, and contrasted with morphologically similar species.

## ﻿Material and methods

### ﻿Study area

Specimens of the new species were found in the Cajanuma Range, part of the Podocarpus National Park (PNP) in the province of Loja, southern Ecuador, and in two areas located in the department of Amazonas, northern Peru: The rural community of Diosan, located in the district of Granada, province of Chachapoyas, and the Área de Conservación Privada Huaylla Belén-Colcamar (hereafter ACPHBC), located in the district of Colcamar, province of Luya.

### ﻿Fieldwork and taxonomic comparison

Specimens of the new species were collected during fieldwork conducted in the two Peruvian study areas between 2018 and 2024 under the respective authorizations for scientific collecting (N° AUT-IFL-2018-025 and N° AUT-IFL-2023-076). Specimens were pressed, dried at 45 °C in an electric oven, labeled, and deposited in the herbarium of the Universidad Nacional Toribio Rodríguez de Mendoza de Amazonas (KUELAP). Flowers from both areas were preserved in a solution of 70% ethanol with 1% glycerol, examined, and measured under a stereomicroscope. Prior to pressing, one plant from each locality was photographed in situ with a digital camera, and Lankester composite dissection plates (LCDP; Karremans 2020) were prepared with Adobe Photoshop v. 24.1.0.

A revision of the specialized literature, including taxonomic descriptions of previously known Andean species of Pleurothallis subgenus Pleurothallis section Macrophyllae-Fasciculatae, was conducted. Specimens of the genus *Pleurothallis* were examined in the herbaria AMES, AMO, F, GH, HOXA, K, KUELAP, MEXU, MO, MOL, NY, QCE, QCNE, US, and USM, during which one additional record of the new species (two duplicate sheets) previously collected in Ecuador was found at MO. A detailed comparison with similar species in the *P. cardiostola–P. lilijae* complex was conducted, and their distinguishing features were evaluated (Table 1, Fig. 4).

**Table 1.** Species of the morphologically defined *P. cardiostola*-*P. lilijae* complex.

*Pleurothallis adelphe* Luer & Hirtz, Lindleyana 11(3): 142--143, f. 2. (1996)
*Pleurothallis alopex* Luer, Selbyana 3(1--2): 46 (1976)
*Pleurothallis andreaskayi* Mark Wilson & B.T. Larsen, Havard Papers in Botany 27(2): 196 (2022)
*Pleurothallis apopsis* Luer, Selbyana 5(2): 160. (1979)
*Pleurothallis barrowii* Schuit., Orchideen Journal 25(2): 55--57 (2018)
*Pleurothallis bilobulata* M.M.Jiménez, Ocupa & Vélez-Abarca, Phytotaxa 518(1): 079--086 (2021)
*Pleurothallis cardiostola* Rchb.f., Bonplandia (Hannover) 2: 26 (1854)
*Pleurothallis carmensotoana* Mark Wilson & B.T. Larsen, Harvard Papers in Botany 27(2): 201 (2022)
*Pleurothallis carrenoi* Carnevali & I. Ramírez, Ernstia no. 44: 18 (-20), fig (1987)
*Pleurothallis castanea* Mark Wilson, G. Merino & J. D. Werner, Lankesteriana 16(3): 358, figs. 2A, 9--10 (2016)
*Pleurothallis compressa* Luer, Lindleyana 11: 75 (1996)
*Pleurothallis culpameae* (Luer) J.M.H.Shaw, Orchid Review 122: 76 (2014)
*Pleurothallis diabolica* Luer & R. Escobar, Orquideología 14(2): 142 (1981)
*Pleurothallis diazii* (Luer & Endara) J.M.H.Shaw, Orchid Review 122: 76 (2014)
*Pleurothallis dilemma* Luer, Revista Soc. Boliv. Bot. 3: 45 (2001)
*Pleurothallis gonzaloi* J.S. Moreno, Rinc.-González & Gal.-Tar., Harvard Papers in Botany 27(2): 210 (2022)
*Pleurothallis labajosii* Revatta-Bustos, Edquén & J.P.Arista (this paper)
*Pleurothallis lanigera* Luer & Hirtz, Lindleyana 3(3): 146 (145, fig.) (1988)
*Pleurothallis lilijae* Foldats, Acta Bot. Venez. 3: 379 (1968)
*Pleurothallis mahechae* J.S. Moreno, Sierra-Ariza & L.C. Pina, Harvard Papers in Botany 27(2): 213 (2022)
*Pleurothallis neobarbosae* J.M.H.Shaw, Orchid Review 122: 77 (2014)
*Pleurothallis ortegae* Luer & Hirtz, Lindleyana 11: 174 (1996)
*Pleurothallis peculiaris* Luer, Selbyana 3: 158 (1976)
*Pleurothallis penelops* Luer, Selbyana 2: 387 (1978)
*Pleurothallis perijaensis* Dunst., Selbyana 2: 210 (1978)
*Pleurothallis perryi* Luer, Selbyana 5: 174 (1979)
*Pleurothallis puyoensis* Criollo, Journal of the Botanical Research Institute of Texas 19(2): 90 (2025)
*Pleurothallis ramiromedinae* Thoerle & Hirtz, Orchideen Journal 6(1): 6 (2018)
*Pleurothallis rikseniana* Mark Wilson & B.T. Larsen, Harvard Papers in Botany 27(2): 208 (2022)
*Pleurothallis sabanillae* M.M.Jiménez & Vélez-Abarca, Phytotaxa 607(3): 171 (2023)
*Pleurothallis troglodytes* Luer, Selbyana 7: 125 (1982)
*Pleurothallis tobarii* (Luer & Hirtz) Pfahl, Internet Orchid Sp. Photo Encycl. Nomencl. Notes 1(3a): 1 (2012)
*Pleurothallis valladolidensis* Luer, Phytologia 54: 388 (1983)
*Pleurothallis volans* Luer & Hirtz, Lindleyana 11: 195 (1996)
*Pleurothallis whitteniana* Mark Wilson & B.T. Larsen, Harvard Paper in Botany 27(2): 201 (2022)

The overall description of the new species was based on all four Peruvian collections and the Ecuadorian one located at MO; measurements of floral parts were taken from alcohol-preserved flowers of all Peruvian collections.

### ﻿Conservation status

The conservation status of the new species was assessed using the IUCN Red List Categories and Criteria (IUCN Standards and Petitions Committee 2024). The extent of occurrence (EOO) and area of occupancy (AOO) were determined using the online tool GEOCAT (Geospatial Conservation Assessment Tool; Bachman et al. 2011), with the default cell width of 2 km.

## ﻿Taxonomic treatment

### ﻿ Pleurothallis labajosii

Taxon classificationPlantaeAsparagalesOrchidaceae

Revatta-Bustos, Edquén & J.P.Arista sp. nov.

7A54255E-CE53-5EA2-B433-800FE1799BCF

urn:lsid:ipni.org:names:77369125-1

Figs 2, 3C–H

#### Type.

Peru • Amazonas: Luya, Colcamar, Área de Conservación Privada Huaylla Belén – Colcamar, sector Yacuchinga, -6.301636, -78.012617, 3329 m elevation, 23 February 2024, *F. Revatta-Bustos et al. 075* (Holotype: KUELAP!).

#### Diagnosis.

Similar to *P. alopex* Luer, differing from the latter in the linear to narrowly lanceolate leaves (vs. lanceolate); reclining spathaceous bract (vs. erect spathaceous bract); pale yellow to brownish flowers (vs. burgundy); flower resupinate, facing away from leaf (vs. flower non-resupinate, facing toward leaf under spathaceous bract); dorsal sepal reflexed from the middle (vs. dorsal sepal not reflexed); synsepal often deeply reflexed from the base (vs. not reflexed).

#### Description.

Epiphytic, caespitose, suberect to arcuate ***herb*** 30−50 cm in height. ***Roots*** simple, cylindrical, whitish, flexuous, to 15 cm long, 0.75−1.25 mm in diameter. ***Stems*** straight to slightly arcuate, decurved at the apex, 1-leaved, terete, 18.5−32 × 0.15−0.29 cm, partially covered by two narrowly tubular, obtuse, pale-to-dark maroon sheaths, these rugose along the veins, 1.8−7.4 cm long. ***Leaf*** pendent, dull green, oblong-lanceolate, 19−27 × 1.2−2 cm, apex acute-attenuate, base cordate, somewhat conduplicate, basal lobules rounded, partially imbricating, upper surface of the apical half of the leaf pubescent, the trichomes simple, narrowly conical, translucent. ***Compound inflorescence*** producing several single-flowered coflorescences, 1−2 having open flowers at a time; peduncle abbreviated, completely covered by two overlapping, chartaceous bracts, these conduplicate, obliquely funnelform, rounded and apiculate at apex, glabrous to densely papillose, the papillae arranged in longitudinal rows, 11−22 mm long. ***Floral bracts*** tubular-funnelform, membranaceous, obtuse, and apiculate, up to 12 mm long. ***Pedicel*** terete, attenuating slightly towards the base, greenish-white, 15−18 × 1.12−1.24 mm. ***Ovary*** terete, slightly arcuate, olive green to reddish maroon, with black dots, 6-sulcate 8−9 × 1.88−2.07 mm. ***Flowers*** resupinate, fleshy, sepals and petals pale yellow, reddish maroon, or brownish with purplish veins; lip orange to orangish-maroon, sometimes with the basal excavation purple; column dull white to yellow. ***Dorsal sepal*** recurved above the middle, elliptic to elliptic-ovate, obtuse, sometimes shortly apiculate, 5-veined, adaxially minutely papillose, 12.3−15.0 × 10−10.3 mm. ***Lateral sepals*** connate throughout their length, forming a convex, ovate to ovate-triangular, narrowly rounded synsepal often reflexed from the base, with recurved margins, 4-veined, adaxially minutely papillose, 12.5−15 × 6.7−9.3 mm. ***Petals*** recurved, obliquely lanceolate, shortly acuminate, 1-veined, adaxially papillose, margins irregularly ciliate, 10.8−11.4 × 2.8−3.0 mm. ***Lip*** sessile, entire, ovate-pandurate, with a triangular, minutely papillose excavation occupying its basal 2/3, and a deeper, rounded, lustrous central area (glenion), 3-veined, lateral margins recurved, apex rounded, base provided at each side with a rounded, retrorse auricle, the auricles partially surrounding the column, 4.5−5.9 × 3.5−5 mm. ***Column*** short, straight, subconical, truncate, somewhat compressed dorsiventrally, 2.5−3.5 mm long, ca. 3 mm wide at the base, very pale yellow or cream colored. ***Anther*** yellow, subovoid-quadrangular, apex truncate, base cordate, 1.2 × 1 mm. ***Pollinarium*** formed by two narrowly ovoid pollinia; these slightly incurved, lustrous, deep yellow, somewhat translucent, ca. 1.4 mm long, united at the apex to a single, globose, whitish viscidium. ***Rostellum*** linear. ***Stigma*** bilobate, with the receptive surfaces rounded, one at each side of the anther. ***Capsule*** not seen mature; developing capsule cylindrical-fusiform, ca. 1.9 × 3 mm.

#### Etymology.

The specific epithet honors Mr. Darío Labajos Canlla, a member of the rural community of Diosan, Granada, in recognition of his devoted efforts towards the conservation of the montane forests of the region.

#### Distribution and habitat.

**Figure 1.**
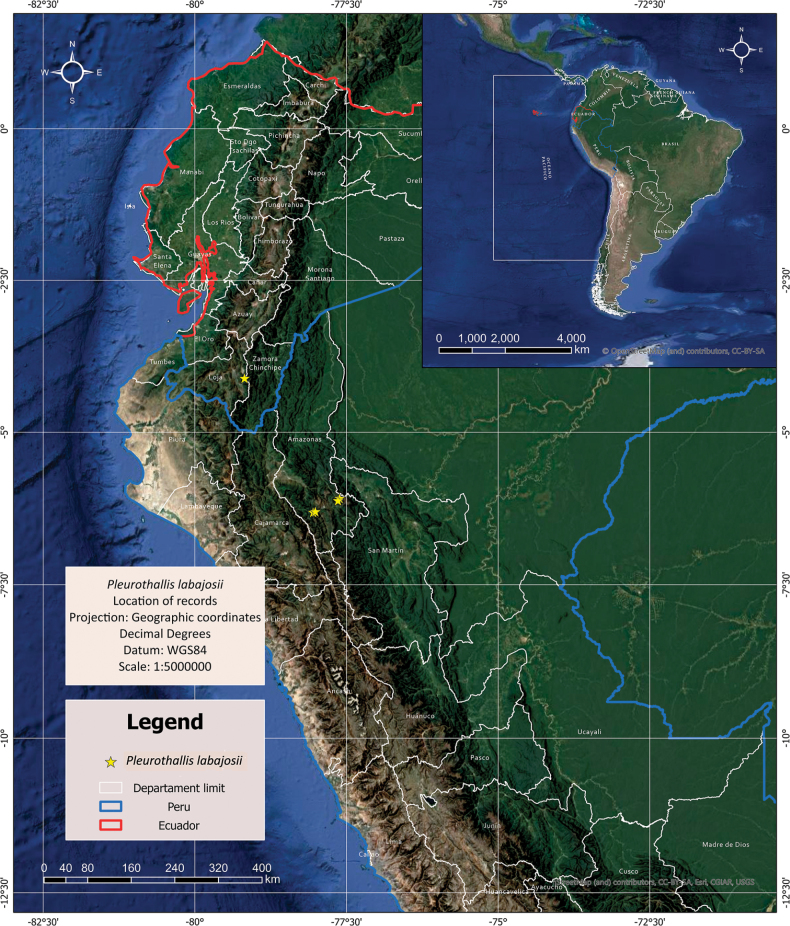
Known distribution of *Pleurothallis labajosii* (map prepared by Julio Puscan Rojas).

Known only from the Eastern Andean Ridge in southern Ecuador and northern Peru (Fig. 1). Epiphytic or less commonly terrestrial on leaf litter, in upper montane cloud forest (*sensu*Kappelle 2004; Fig. 3A–C) at 2,850−3,330 m elevation. In Diosan, the most conspicuous floristic elements of the forest are *Weinmannia* sp., *Berberis* sp., *Chusquea* sp., and Blechnum aff. auratum (Fée) R.M.Tryon & Stolze, whereas in the ACPHBC, *Columellia oblonga* Ruiz & Pav. and *Weinmannia* sp. are common, and both are phorophytes of *P. labajosii*.

**Figure 2.**
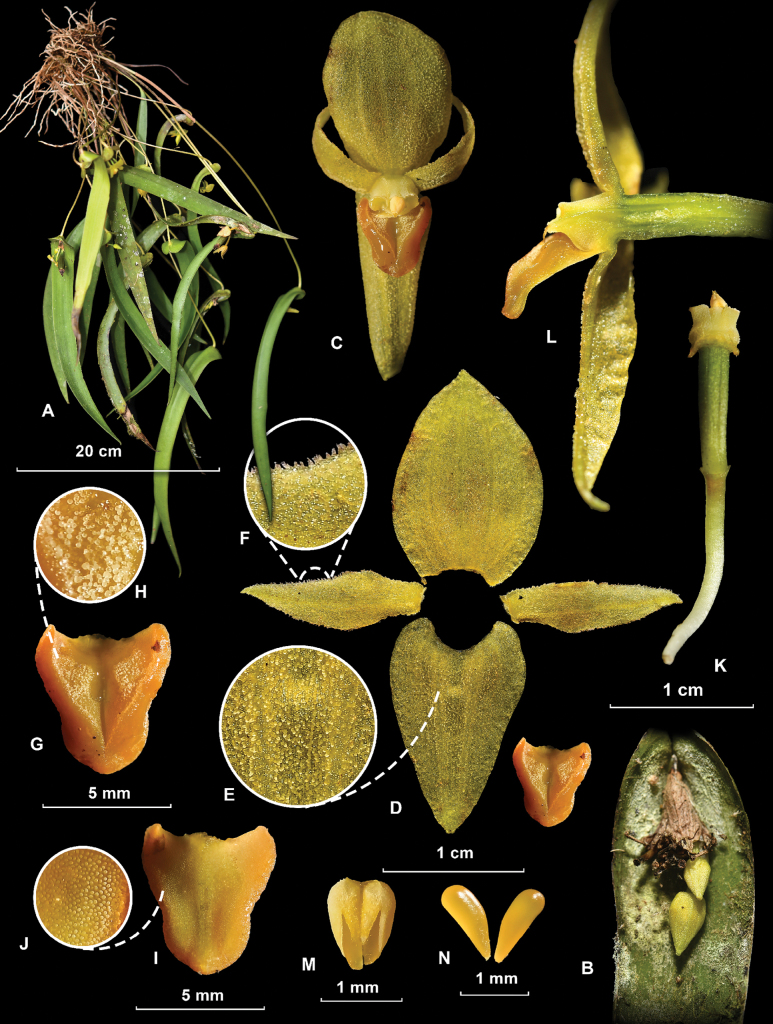
*Pleurothallis labajosii*. A. habit; B. base of leaf and coflorescence; C. flower from front; D. dissection of the perianth; E. close-up of the papillae on the inner surface of the synsepal; F. close-up of the papillae on the inner surface of the petals; G. labellum from above; H. close-up of the papillae on the inner surface of the labellum; I. labellum from below; J. close-up of the papillae on the outer surface of the labellum; K. Pedicel, ovary, and column from above; L. Longitudinal section of flower; M. anther from below; N. pollinia (photographs from the type by José D. Edquén; plate prepared by Elmer Yrigoín).

**Figure 3.**
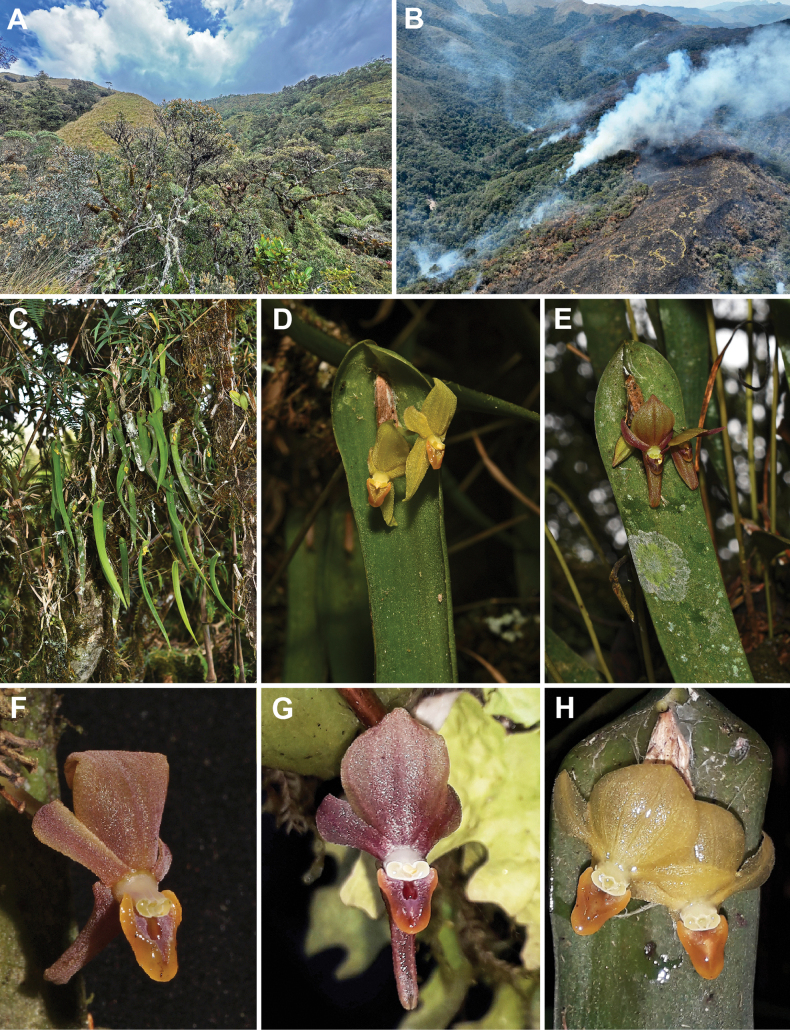
*Pleurothallis labajosii*. A. upper montane cloud forest at the Área de Conservación Privada Huaylla-Belén-Colcamar (ACPHBC); B. forest fire in the ACPHBC in September 2024; C. flowering plant *in situ* at the ACPHBC; D, E. two flower color variants at the ACPHBC; F. flower of a plant from Diosan; G, H. two flower color variants at the Cajanuma Range, Podocarpus National Park (Photographs A–F by José D. Edquén, G–H by Jaime Peña).

#### Phenology.

Flowering throughout the year. Developing fruits recorded in January and February.

#### Conservation assessment.

*Pleurothallis labajosii* is known from five locations backed up by verifiable specimens, two of them in the ACPHBC, two in Diosan, and one in PNP. The EOO estimated by Geocat is 7,561.02 km^2^, whereas the AOO is 20 km^2^. In the ACPHBC, 67 mature individuals were recorded originally in five lineal transects 1 km long and 5 m wide, but approximately 30% of the habitat of this species adjacent to the Huaylla Belén river was devastated by a forest fire in September 2024, resulting in the probable loss of 45 individuals (67%; Fig. 3B). It is worth noting that, in spite of the ACPHBC being under nominal protection in the figure of Área de Conservación Privada (Private Conservation Area), the lack of strict control makes the area vulnerable to deforestation for expanding livestock grazing, a persistent threat to the remaining populations. On the other hand, in Diosan the two populations consisted of five and 12 individuals, respectively, both restricted to relict forest patches and threatened by deforestation for livestock grazing, and one of them also by the opening of a road connecting the town of Granada with the Siete Lagunas sector, an area of ecotouristic potential. No information is available on the status of the population of the Podocarpus National Park, but the several photographic records from the area available in iNaturalist (see below) suggest that it is not uncommon there. However, the limited information on the distribution and the population features of this species does not permit an objective assessment of its conservation status, and we suggest its inclusion in the category of “Data deficient” (DD; IUCN Standards and Petitions Committee 2024).

#### Additional specimens examined.

**Ecuador. “Zamora-Chinchipe” [Loja**]: • Cajanuma Range, south of Loja [approximate coordinates: -4.112756, -79.180501], 2750 m, epiphytic in cloud forest, 21 March 1985, *C. Luer et al. 10741* (MO, ×2). **Peru. Amazonas**: • Chachapoyas, Granada, comunidad campesina de Diosan, bosques relictos, puente Canchi camino a Siete Lagunas, -6.121617, -77.639594, 3054 m, 13 August 2018, *J.D. Edquén 2541* (KUELAP!); • Luya, Colcamar, Área de Conservación Privada Huaylla Belén–Colcamar, sector Yacuchinga, -6.313064, -78.048867, 2858 m, 10 January 2024, *F. Revatta-Bustos et al. 016* (KUELAP!); • Luya, Colcamar, Área de Conservación Privada Huaylla Belén–Colcamar, sector Yacuchinga, -6.08495, -77.613439 lon, 3207 m, 15 July 2018, *J.D. Edquén 2540* (KUELAP!).

#### Other records.

**Ecuador. Loja**: Cajanuma Range, Podocarpus National Park, January 2023, observation in iNaturalist by Nolan Exe, https://www.inaturalist.org/observations/157376955 (accessed 16 Aug 2025); Reserva Privada Madrigal del Podocarpus, 29 April 2023, observation in iNaturalist by Amarú Ramón Salcedo, https://www.inaturalist.org/observations/158473653 (accessed 16 Aug 2025); Reserva Privada Madrigal del Podocarpus, 28 Dec 2023, observation in iNaturalist by Florent Pouzet, https://www.inaturalist.org/observations/195265250 (accessed 16 Aug 2025); Refugio Cajanuma, Parque Nacional Podocarpus, observation in iNaturalist by Belen Montenegro, https://www.inaturalist.org/observations/21883479 (accessed 16 Aug 2025); Parque Nacional Podocarpus, photos by Jaime Peña privately shared with M. Wilson (reproduced in Fig. 3G, H).

##### ﻿Taxonomic discussion

**Figure 4.**
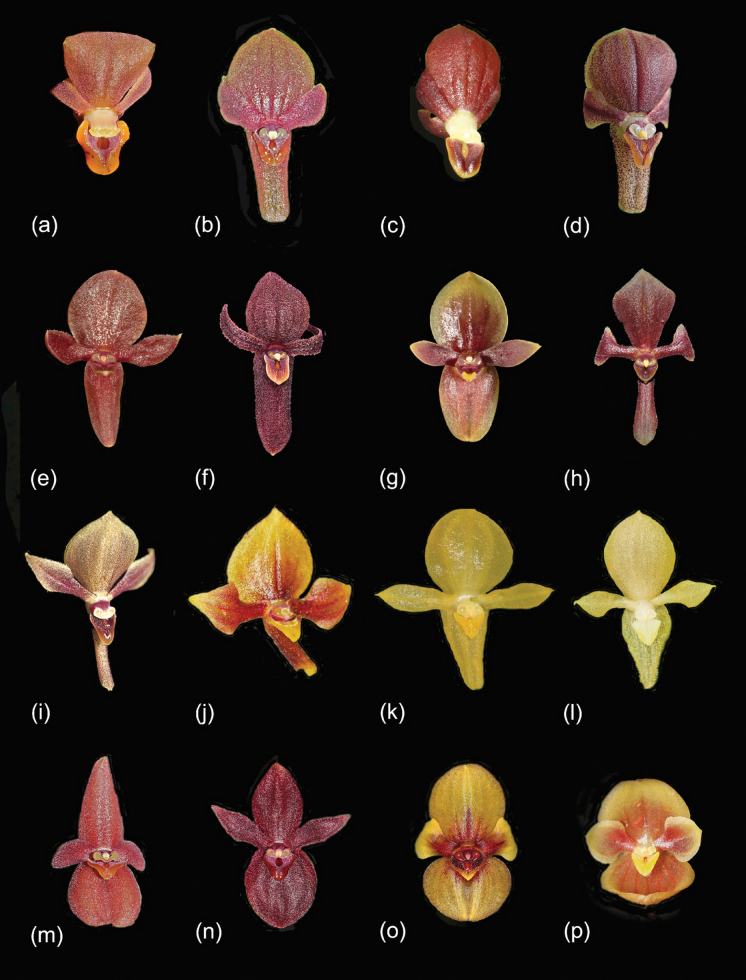
Selected species in the *Pleurothallis cardiostola*-*P. lilijae* complex with resemblance to *P. labajosii* (most similar to less similar; not to scale): a. *P. labajosii* (photo by José D. Edquén); b. *P. alopex* (photo by Alfredo Fuentes); c. *P. penelops* (photo by Duane McDowell); d. *P. gonzaloi* (photo by Sebastian Moreno); e. *P. carmensotoana* (photo by Mark Wilson); f. *P. mahechae* (photo by Sebastian Moreno); g. *P. whitteniana* (photo by Mark Wilson); h. *P. sabanillae* (photo by Marco Jiménez); i. *P. lilijae* (photo by Sebastian Moreno); j. *P. tobarii* (photo by Francisco Tobar); k. *P. apopsis* (photo by Francisco Tobar); l. *P. andreaskayi* (photo by Mark Wilson); m. *P. cardiostola* (photo by Alan Gregg); n. *P. lanigera* (photo by Kevin Holcomb); o. *P. ortegae* (photo by Ron Parsons); and p. *P. volans* (photo by John Varigos).

The *Pleurothallis cardiostola–Pleurothallis lilijae* complex, as defined by Wilson et al. (2022), is a group of species within section Macrophyllae-Fasciculatae (Table 1, Fig. 4). The members of the group were assigned subjectively based on the possession of a subset of shared morphological traits. Wilson et al. (2022) refrained from resurrecting subsection Cardiostolae (Luer 1988) for this group until phylogenetic or phylogenomic analyses indicate that this is warranted. Preliminary phylogenetic analysis of nuclear internal transcribed spacer (nrITS) sequences (Wilson et al. 2011) did not, in fact, support segregation of these species from *Macrophyllae-Fasciculatae*, but nrITS sequence variation in this group is very low and inadequate for such analyses. It is hoped that ongoing phylogenomic analyses (T. Arias, L.A. Eserman, and M. Wilson, unpublished data) will facilitate a robust and stable infrageneric classification of *Pleurothallis* and indicate whether the *P. cardiostola–P. lilijae* species complex represents a separate genetic lineage from other members of *Macrophyllae-Fasciculatae* and, hence, whether they should be assigned to a different taxonomic group.

The complex as currently recognized, including the recently published *P. sabanillae* M.M.Jiménez & Vélez-Abarca (Jiménez et al. 2023) and *P. puyoensis* Criollo (Cornejo and Criollo-Naula 2025), plus the species described herein, consists of 35 species (Table 1). Other taxonomists have suggested additional members of the complex, but until comprehensive phylogenomic analyses are conducted, the composition of the list remains subjective. The complex is distributed from Venezuela south to Bolivia and Paraguay, and it is possible that “true” *P. lilijae* occurs throughout that distribution, but herbarium-based distribution records are unreliable due to frequent misidentifications. Other species are much more narrowly distributed, including the present species, which appears to be limited to southern Ecuador and northern Peru.

While a set of shared traits unites these species into a complex, the subset of traits and variation in those traits allow us to distinguish between them. Distinguishing characteristics include: leaf (ovate, wide-lanceolate, or narrowly lanceolate; trichomatous or glabrous); leaf basal lobes (incurved or not incurved; imbricating or not); spathaceous bract (reclining or erect); flower (resupinate or non-resupinate); dorsal sepal (partially reflexed from midpoint or not reflexed); synsepal (deeply reflexed from base or not reflexed; if not reflexed, rolled or extended); lip position (angle between lip and synsepal); petal and sepal indumentum (trichomatous, papillose, or glabrous); and lip morphology (triangular cavity with pronounced glenion at base or lip convex with ornamentation or calli). These floral traits alone are sufficient to distinguish many of the species (Fig. 4) and, when supplemented by other vegetative traits, allow relatively easy identification of most species.

We compare *Pleurothallis labajosii* to *P. alopex* (Fig. 4B) as the most similar species, but the observations in iNaturalist from PNP (listed above) were all misidentified as *P. penelops* Luer (Fig. 4C), a species from farther north near Paute, Morona-Santiago, Ecuador. The feature causing confusion between *P. labajosii* and *P. penelops* is the synsepal, which in both cases is strongly reflexed from the base, such that in front view, the synsepal may not be visible. However, *P. labajosii* is easily distinguished from *P. penelops* by the variable flower color, from yellow to reddish maroon or brownish with purplish veins, lip orange to orangish maroon (vs. uniformly very dark, intense burgundy); column color very pale yellow or cream colored (vs. white); and petals and sepals papillose (vs. glabrous). *Pleurothallis labajosii* is also somewhat similar to *P. gonzaloi* J.S.Moreno, Rinc.-González & Gal.-Tar. (Fig. 4D) from Colombia but can be distinguished by the papillose dorsal sepal (vs. glabrous); very pale yellow or cream-colored column (vs. cream suffused with red-purple at base); and ovate-pandurate lip with auricles around the column (vs. triangular with no auricles). Other species in the group (Fig. 4E–P) are much less likely to be confused with *P. labajosii*.

## Supplementary Material

XML Treatment for Pleurothallis labajosii

## ﻿Additional information

### Use of AI

No use of AI was reported.

### Author ORCIDs

Fiorela Revatta-Bustos https://orcid.org/0009-0004-9020-0520

José D. Edquén https://orcid.org/0000-0002-7289-3974

Jessy P. Arista https://orcid.org/0000-0001-8334-5538

Elmer Yrigoín https://orcid.org/0000-0002-0517-462X

Rosalynn Yohanna Rivera López https://orcid.org/0000-0002-8552-8634

Mark Wilson https://orcid.org/0000-0003-3044-7471

Mabel Enco https://orcid.org/0009-0003-6105-9313

Kely Edquen https://orcid.org/0009-0007-4768-7259

Santos Leiva-Espinoza https://orcid.org/0000-0003-1710-1994

Manuel Oliva-Cruz https://orcid.org/0000-0002-9670-0970

Gerardo A. Salazar https://orcid.org/0000-0002-5203-5374

### Data availability

All of the data that support the findings of this study are available in the main text.
